# A Time-Dependent Vehicle Routing Problem for Instant Delivery Based on Memetic Algorithm

**DOI:** 10.1155/2022/5099008

**Published:** 2022-08-10

**Authors:** Shuxian Cui, Qian Sun, Qian Zhang

**Affiliations:** School of E-business and Logistics, Beijing Technology and Business University, Beijing 100048, China

## Abstract

Instant delivery is an intermediary bridge for same-city O2O services and an important part of urban short-distance logistics. The route planning and scheduling of instant delivery need to balance cost and customer satisfaction and consider the impact of traffic conditions on the distribution process. In this paper, we propose a vehicle routing problem model considering two types of customer time windows under time-dependent road networks and design a memetic algorithm combined with genetic algorithm and variable neighborhood search to solve the problem. By comparing the results of the different time periods and conducting sensitivity analysis for the two types of customer time windows, the effectiveness of the model and algorithm is verified.

## 1. Introduction

Instant delivery is a short-distance logistics activity in the same city that connects online and offline logistics, spawned by O2O near-field consumption behavior. Compared with traditional delivery, instant delivery has the advantages of quick response, strong timeliness, greater influence by driving road conditions, and better communication with customers. It has the characteristics of close contact and so on [1]. With the continuous growth of the demand for instant delivery, the conflict between the complex driving environment and the high delivery time is further intensified. The morning and evening peak traffic congestion have a great impact on the speed of instant delivery. Therefore, considering the external driving environment factors in maintaining high-quality delivery services while effectively reducing delivery costs has become a new focus in the development of instant delivery business.

Reasonable path planning scheme can effectively improve delivery efficiency, so ongoing research is focusing on the vehicle routing problem for instant delivery platforms [2]. In previous related studies, Yu et al. [3] constructed an instant delivery route optimization model based on customer classification for the instant delivery platform and designed an improved genetic algorithm to solve it. Shi et al. [4] considered the situation in the case of unreasonable capacity allocation where riders who provide O2O services can provide delivery services for B2C merchants in their spare time, and a joint pickup and delivery path model is established on this basis. Gu et al. [5] designed a collaborative instant delivery scheme with traditional vehicles and unmanned aerial vehicles, divided the delivery process into two stages, and established models according to the characteristics of the delivery activities in each stage. Huang et al. [6] proposed a delayed scheduling strategy, and a two-stage scheduling optimization model was constructed with the goal of minimizing the amount of overtime tasks. Reyes et al. [7] proposed the problem of instant delivery food delivery route as the basic form of dynamic delivery problem. Zhao et al. [8] considered the instant distribution problem against the background of the new retail front-end warehouse, and the optimal scheduling of vehicles was transformed into the shortest path problem in the space-time network by using the space-time network modeling method. Dai et al. [9] studied the capacity coordination in O2O instant distribution in order to solve the problem of order allocation and route planning in the distribution situation, an optimization model was established, and the rolling optimization method was used to solve it. Tao et al. [10] established an optimization model combining order allocation and path planning for the instant delivery vehicle routing problem.

Customer satisfaction is an important factor that needs to be considered in instant delivery vehicle route problem [11]. In previous studies of vehicle routing problem considering customer satisfaction, Chen et al. [12] proposed an optimization model suitable for catering, which characterized customer satisfaction through the rider's arrival time function, and maximized customer time satisfaction as the optimization objective; the rationality of the model was verified by simulation experiments. Wang et al. [13] considered the randomness of travel time in food delivery activities, proposed and verified that the travel time of riders between two demand points obeys a normality distribution, and established an optimization model aiming at maximizing customer satisfaction. Zhang et al. [14] introduced the customer service priority in the O2O takeaway delivery problem, considered customer satisfaction and delivery cost, and established a model considering customer satisfaction with service priority and travel cost. Jin et al. [15] introduced the concept of order delivery efficiency during the noon ordering peak, a mixed integer programming model for rider distribution route optimization was constructed with the goal of maximizing transportation efficiency, and an improved iterative local search algorithm was designed to solve the model. Liao et al. [16] considered the situation of new order merging after the delivery plan is set in the green vehicle routing problem, and proposed a multi-objective optimization model aiming at the highest customer satisfaction, the least carbon emissions, and the least transportation capacity.

Intelligent optimization algorithms have good performance in solving optimization problems such as location problem, order allocation, job-shop scheduling problem, and vehicle routing problem. Zhang et al. [17] proposed an improved whale optimization algorithm to solve the electric vehicle charging station location problem. Wang [18] used the sparrow search algorithm to improve traditional backpropagation neural network and applied the algorithm to evaluate the manufacturing capacity of smart job-shop. Liu et al. [19] proposed an improved genetic algorithm to solve the vehicle routing problem of cargo transport O2O platforms. Kunnapapdeelert and Thawnern [20] introduced saving algorithm to solve the capacitated vehicle routing problem. The ability of intelligent optimization algorithms depends on the breadth and depth of search process. Due to the combination of global search algorithm and local search strategy, the memetic algorithm shows strong search efficiency in solving the vehicle routing problem. Labadi et al. [21] used a memetic algorithm composed of genetic algorithm and four local search operators such as 2-opt to verify the rationality of the algorithm in solving the TDVRP. Mendoza et al. [22] used a local search strategy combined with a genetic algorithm to solve the multi-compartment vehicle routing problem with uncertain demand and designed experiments to show that the algorithm has good performance. Zhang et al. [23] proposed a memetic algorithm combining guided ejection search and boundary crossing strategy for the vehicle routing problem of pickup and delivery in reverse logistics, operated on the offspring through a variety of repair and education operators, and verified that the algorithm has good performance by comparing it with a variety of other algorithms.

The above research provides a certain theoretical basis for the vehicle routing problem in instant distribution activities and the measurement of customer satisfaction in the problem, but most of the studies do not consider the external environment influence, especially the very regular urban traffic congestion peak which has an impact on the distribution speed, and ignore whether the customer specifies the delivery time in the actual scenario. In addition, this paper considers two forms of customer satisfaction in the instant delivery scenario and divides the time-dependent road network according to different traffic congestion levels. The memetic algorithm of variable domain strategy is used to solve the proposed model.

## 2. Model Building

### 2.1. Problem Description

An instant delivery platform has released a series of pickup and delivery tasks. The riders who receive the tasks need to pick up the goods at different pickup points and send them to the corresponding receiving points. During this process, each pickup and delivery demand should be held accountable. The rider needs to complete the delivery task within the time window expected by the customer at each receiving point. At the same time, the customer expected time at the receiving point has two types: specified time and unspecified time. Multiple orders can be serviced per vehicle, but each order is serviced by one and only one vehicle. The platform needs to reasonably arrange the order and service sequence of each vehicle service, take into account the delivery cost while meeting the customer's expected delivery time, and formulate a routing planning scheme with the least cost.

Make the following assumptions based on the problem:

1. An order must be picked up before delivery occurs.
2. The pickup and delivery tasks of the same order need to be completed by the same vehicle.
3. There is no service time constraint at the pickup point.
4. Vehicles have different speeds at different real-time moments.

### 2.2. Mathematical Model

The variables and parameters required to build the model are defined as follows:

  K: The set of delivery vehicles, *K*={*k*}, *k*=1,2,3,….
  C: The set of delivery points, *C*={*n*}, *n*=1,2,3,….
  P: The set of pickup points, *P*={*m*}, *m*=*n*+1, *n*+2, *n*+3….

Define the set of all pickup and delivery nodes as N, *N*=*C* ∪ *P*.

According to the nature of the order, the pickup point and delivery point of each order should correspond to each other, so the delivery point and pickup point of the same order are defined in the same position in their respective sets.

  R: The set of orders, *R*={*r*}, *r*=1,2,3….
  Q: The maximum vehicle capacity.
 
*C*_*m*_: The unit operating cost of the vehicle.
 
*C*_*n*_: The fixed cost of vehicle.
 
*D*_*ij*_: The distance from node *i* to node j.
 
*d*_*i*_: The delivery demand at node *i*.
 
*p*_*i*_: The pickup demand of node *i*.
  [*e*_*i*_, *l*_*i*_]:The time window for customer *i* at the receiving point.
  [*E*_*i*_, *L*_*i*_]: The earliest and latest time windows that can be tolerated by customer *i* at the receiving point.
 
*T*_*ik*_: The time when the vehicle actually arrives at node *i*.
 
*z*_*ik*_: The capacity of vehicle *k* after serving node *i*.
 
*s*_*i*_: The service time of node i.
 
*t*_*ijk*_: The time it takes for vehicle *k* to travel from node *i* to node *j*.
 
*X*_*ijk*_: If the vehicle travels from the customer point *i* to the customer point *j*, then *X*_*ijk*_=1; otherwise, the value is 0.
 
*Y*_*ik*_: If the customer point is served by vehicle *k*, then *Y*_*ik*_=1; otherwise, the value is 0.
 
*Z*_*rk*_: If order *r* is serviced by vehicle *k*, then *Z*_*rk*_=1; otherwise, the value is 0.
 
*α*: The customer satisfaction penalty cost coefficient.

#### 2.2.1. Time-Dependent Function

**Figure 1:**
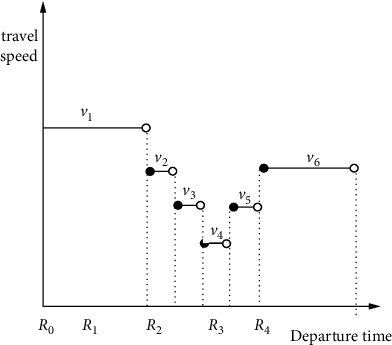
Time-dependent function for uneven periods.

**Figure 2:**
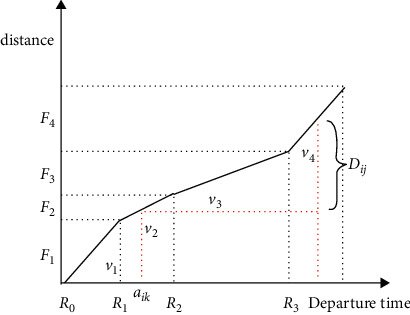
Calculation of travel time between nodes in a time-dependent network.

The construction of the time-dependent function in the existing literature usually adopts the form of driving speed-reality moment proposed by Ichoua, and the time-dependent function in this form follows the “first-in, first-out” criterion [24]. The time-dependent function in most studies divides the time of day into morning peak, evening peak, and peak hours, and each time period corresponds to different driving speeds, or the time is further divided into equal time periods such as 60 minutes and 30 minutes [25–27]. The speed change described by this processing method is slightly different from the actual situation. Therefore, according to the characteristics of the problem, this paper further divides each time period with reference to the speed change in the real situation. In the flat peak period, every 30 minutes is a time period, and in the peak period, every 10 minutes is a time period. It is constant in each time period, and this form of time-dependent function also satisfies the “first-in, first-out” criterion. In a time-dependent network, the time-dependent function for uneven periods is shown in Figure 1, and the total travel time of a vehicle between two points is calculated as shown in Figure 2.

According to the construction principle of time-dependent function, the set of divided time periods is *R*={[*R*_0_, *R*_1_], [*R*_1_, *R*_2_] … [*R*_*o*−1_, *R*_*o*_]},  *o*=0,1,2,3…; the speed in the time period [*R*_*o*−1_, *R*_*o*_] is *v*_*O*_; and the complete distance the vehicle can travel in the time period [*R*_*o*−1_, *R*_*o*_] is *F*_*O*_. Then, the speed set corresponding to the order of the time period is *V*={*v*_1_,*v*_2_ …,*v*_*o*_}, and the distance set is *F*={*F*_1_,*F*_2_ …,*F*_*o*_}. The steps to calculate the total travel time *t*_*ijk*_ between two points are as follows:

  Step 1. Determine the time interval [*R*_*o*−1_, *R*_*o*_] in which the vehicle leaves the last customer point *j*.
  Step 2. If (*R*_*o*_ − *a*_*ik*_)*v*_*o*_ − *D*_*ij*_ ≥ 0, then the calculation ends: *t*_*ijk*_=*D*_*ij*_/*v*_*o*_. Otherwise, go to the next step.
  Step 3. Accumulate the complete distance traveled in the next time period based on the distance traveled until the result is greater than *D*_*ij*_. When (*R*_*o*_ − *a*_*ik*_)*v*_*o*_+*F*_*O*+1_+*F*_*O*+2_+⋯+*F*_*O*+*φ*_ − *D*_*ij*_ ≥ 0 , the calculation ends; the total travel time at this time is *t*_*ijk*_=[(*R*_*o*_ − *a*_*ik*_)*v*_*o*_+*F*_*O*+1_+⋯+*F*_*O*+*φ*_ − *D*_*ij*_]/*v*_*o*+*φ*_ − *a*_*ik*_.

#### 2.2.2. Customer Satisfaction Function

**Figure 3:**
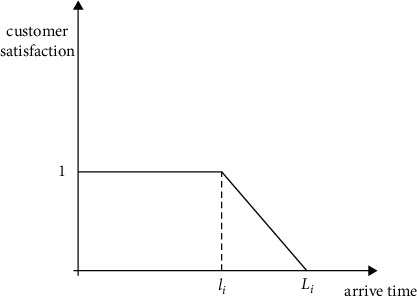
Schematic diagram of A-type customer satisfaction function.

Delivery quality is an important factor affecting satisfaction and reuse intention of customers on O2O delivery platform [28]. In the actual scene of instant delivery, customers have two requirements for delivery time: immediate delivery and delivery within a specified time period. This paper constructs satisfaction functions for customers with different delivery time requirements, as follows:

*(1) Type A of Customer Satisfaction Function*. Type A of customers refers to customers who need to be delivered immediately. In this case, there is no waiting penalty. Delivery within the estimated arriving time *l*_*i*_ will make the customer satisfaction the highest. But when the actual arriving time exceeds *l*_*i*_, the satisfaction function decreases linearly according to the delay time. When the arriving time exceeds the latest time that the customer can tolerate, the customer satisfaction drops to 0. The schematic diagram and expression of the specific satisfaction function of type A customers are shown in Figure 3 and the following formula:

(1) $$\begin{array}{c} S_{a}\left(T_{ik}\right)=\left\{\begin{array}{cc} 1, & \;e_{i}\leq T_{i}\leq l_{i},\\ \frac{t_{i}-l_{i}}{L_{i}-l_{i},} & l_{i}\leq T_{i}\leq L_{i},\\ 0, & T_{i}\geq L_{i}. \end{array}\right. \end{array}$$

**Figure 4:**
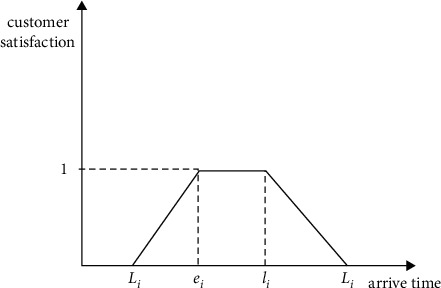
Schematic diagram of B-type customer satisfaction function.

*(2) Type B of Customer Satisfaction Function*. Type B of customers refers to customers who have specified a specific delivery time period. When the arrival time is within the customer's expected time window, customer satisfaction is maximized. When the arrival time is not within the time window but does not exceed the earliest and latest delivery time that the customer can tolerate, the satisfaction function decreases linearly according to the advance and delay time. When the arriving time exceeds the earliest or latest delivery time that the customer can tolerate, the customer satisfaction decreases to 0. The schematic diagram and expression of the specific satisfaction function of type B customers are shown in Figure 4 and the following formula:

(2) $$\begin{array}{c} S_{b}\left(T_{ik}\right)=\left\{\begin{array}{cc} 1, & e_{i}\leq T_{i}\leq l_{i},\\ \frac{T_{i}-E_{i}}{e_{i}-E_{i},} & E_{i}\leq T_{i}\leq e_{i},\\ \frac{T_{i}-l_{i}}{L_{i}-l_{i},} & l_{i}\leq t_{i}\leq L_{i},\\ 0, & T_{i}\geq L_{i},\;T_{i}<E_{i}. \end{array}\right. \end{array}$$

#### 2.2.3. Route Planning Model

In summary, the path planning model with the goal of minimizing the total cost is established as follows:

(3) $$\begin{array}{c} \text{min}f=\sum_{k\in K}\sum_{i\in N}C_{m}Y_{ik}+\sum_{k\in K}\sum_{i\in N}\sum_{j\in N}C_{n}X_{ijk}D_{ij} \end{array}$$

(4) $$\begin{array}{c} =1,\;\forall r\in R, \end{array}$$

(5) $$\begin{array}{c} =1,\;\forall i\in N, \end{array}$$

(6) $$\begin{array}{c} \sum_{i\in N}X_{\text{ijk}}=\sum_{i\in N}X_{\text{jik}},\;\forall j\in N,\;\forall k\in K, \end{array}$$

(7) $$\begin{array}{c} \sum_{i\in N}X_{\text{oik}}\leq 1,\;\forall k\in K, \end{array}$$

(8) $$\begin{array}{c} \sum_{i\in N}X_{i0k}\leq 1,\;\forall k\in K, \end{array}$$

(9) $$\begin{array}{c} \sum_{i\in N}X_{jik}\left(z_{jk}+p_{i}-d_{i}\right)\leq Q,\;\;\forall k\in K, \end{array}$$

(10) $$\begin{array}{c} \sum_{i=0}\sum_{j=0}X_{\text{ijk}}\leq \left|S\right|-1,\;\forall S\subseteq N,\;S\neq \emptyset ,\;\forall k\in K, \end{array}$$

(11) $$\begin{array}{c} , \end{array}$$

(12) $$\begin{array}{c} T_{ik\;}\geq T_{jk},\;\\ i=j,\;\forall i\in C,\;\forall j\in P,\;\forall k\in K, \end{array}$$

(13) $$\begin{array}{c} X_{\text{ijk}}\in \left\{0,1\right\}, \end{array}$$

(14) $$\begin{array}{c} Y_{ik}\in \left\{0,1\right\}, \end{array}$$

(15) $$\begin{array}{c} Z_{rk}\in \left\{0,1\right\}. \end{array}$$

Equation (3) is the objective function that represents the minimum total cost, including the dispatch cost of the vehicle, the travel cost, and the penalty cost for not meeting the customer time window. Equation (4) indicates that each order can be serviced by only one vehicle. Equation (5) indicates that each node is only served once by one vehicle. Equation (6) ensures that the vehicle arrives at and leaves the same node. Equations (7) and (8) indicate that the vehicle can only depart from the station once and return only once. Equation (9) is the constraint of the vehicle load. Equation (10) is used to eliminate sub-loops. Equation (11) represents the relationship between node arrival time, service time, waiting time, and travel time. Equation (12) is the constraint that the delivery time of each order should be greater than the pickup time. Equations (13), (14), and (15) represent the value constraints of decision variables.

## 3. Algorithm Design

Memetic algorithm is also known as the cultural gene algorithm, which was proposed by scholar Pablo Moscato in 1989, and considered to be a kind of hybrid global heuristic search algorithm based on population evolution algorithm [29]. Memetic algorithm does not have a fixed operation process but uses a combination of different global and local search strategies. This combination method can obtain stronger search efficiency than the traditional population optimization algorithm. Therefore, the memetic algorithm has been successfully used in solving various optimization problems. The memetic algorithm designed in this paper mixes the variable neighborhood search strategy on the basis of the genetic algorithm and improves the performance of the algorithm by greedy initialization.

### 3.1. Variable Neighborhood Search

The variable neighborhood search algorithm can expand the search range by continuously changing the neighborhood structure during the search process and obtain a local optimal solution. Therefore, the local search ability of the algorithm can be improved [30]. The process of variable neighborhood search is as follows:

  Step 1. Define *M* neighborhood structures as *N*_*k*_ (*k* = 1, 2, 3,…, *m*).
  Step 2. For the initial solution *S*_0_, use the first neighborhood structure to search. If the new solution *S* is found to be better than *S*_0_, let *S*_0_=*S*, and restart the search from the first neighborhood; if no new optimal solution is found, let *i*+ = 1, and proceed to the next neighborhood structure search.
  Step 3. If *i*≤*m*, repeat Step 2; when the termination condition of the iteration is met, the algorithm ends and the optimal solution is output.

The search ability of the variable neighborhood algorithm depends to a large extent on the design of the neighborhood structure. The neighborhood structure used in this section has two types: inter-path and intra-path.

1. Neighborhood structure between paths. Based on the characteristics of the simultaneous pickup and delivery problem, when performing the neighborhood search between paths, the operator acts on the order instead of a single node; that is, when a node is selected, its corresponding pickup point or delivery point should also be selected at the same time.
2. Relocation operator: randomly select an order from one route, and randomly insert it into another route, where the delivery point should be inserted after the pickup point.
3. Neighborhood structure within the path. Insertion operator: randomly select a node in the path. If this node is a pickup point, it will randomly insert the position before the corresponding delivery point. If this node is a delivery point, it will randomly insert the position after the corresponding pickup point.
4. Swap operator: Randomly select two orders in the path and exchange the order pickup point and delivery point, respectively.

The number of neighborhood searches of the variable neighborhood algorithm will affect the running time of the algorithm to a certain extent. In order to balance the search performance and running time of the algorithm, this paper sets the termination condition of the variable neighborhood search as the maximum algebra that the optimal solution does not change. That is, in the process of variable neighborhood search, if the optimal solution does not change during the generation, the process of variable neighborhood search stops and the current optimal solution is output.

### 3.2. Memetic Algorithm

**Figure 5:**
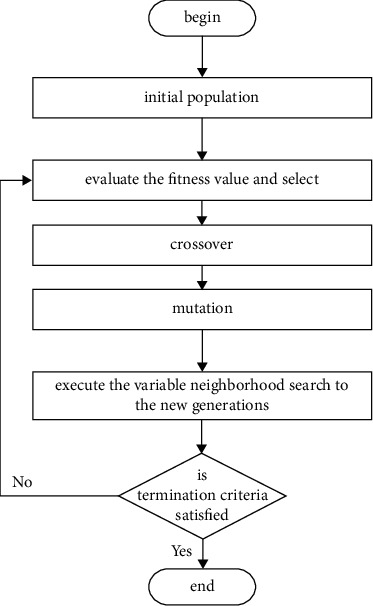
Flowchart of memetic algorithm.

Based on the genetic algorithm, this section uses the greedy algorithm to generate the initial solution and the variable neighborhood strategy to construct a memetic algorithm to effectively solve the TDVRPPDTW problem. The flowchart of the algorithm key steps is shown in Figure 5:

1. Decoding and encoding. In this paper, the natural number encoding method is selected to describe the path. The pickup point is represented by a natural number in [1, *n*], and the corresponding delivery point is represented by a natural number in [*n* + 1, 2*n*]; the delivery point number corresponding to the pickup point *i* is *n* + *i*. In the solution scheme, “0” is used as the dividing point of the driving scheme, and the order of numbers is the order in which the vehicles visit the nodes.
2. Initial population generation. According to the principle of the shortest distance between nodes, the greedy algorithm is used to generate the initial solution, the path rationalization check is carried out on the individual initial solution by determining whether all the delivery points are in the same path of the corresponding pickup points, and the delivery point is always after the corresponding pickup point. If the above two constraints are not met, the unreasonable delivery point position will be transferred to the tail position in the same path as the pickup point.
3. Genetic algorithm process. The selection operations in this section are implemented using the roulette wheel. To maintain the rationality of the path, the OX crossover method is selected to complete the crossover operation during the crossover process. The mutation operation process in the genetic algorithm selects the reverse mutation method.
4. Variable neighborhood search. Perform the variable neighborhood search operation described in 3.1 on all the offspring generated by crossover and mutation in the genetic algorithm.
5. Termination conditions. Judge whether the algorithm reaches the maximum number of iterations; if not, go to Step 3; otherwise, stop the iteration and output the optimal solution.

## 4. Example Analysis

The experimental example in this section is modified on the basis of the example in the literature [12]. The example includes a distribution center and 30 customer nodes, and the coordinates of the customer points are randomly distributed. According to the actual customer distribution, delivery distance, and customer time window of instant delivery, the specific data of the modified calculation example is shown in Table 1. In order to match the actual delivery scenario of instant delivery as much as possible, the following data are supplemented:

1. According to the different types of customer point time windows, the customer points whose left end of the time window is 0 in the calculation example are set as A-type customers, and the rest are B-type customers.
2. According to the actual situation of instant delivery activities, the longest service time in a round of delivery activities is set to 60 minutes, and the service time of the distribution center set in the experiment is [12:00–22:00].
3. According to the law of urban traffic congestion, 17:00–19:00 is set as the evening peak period, and other periods in the calculation example are flat peaks.
4. According to the actual situation, the maximum speed of the delivery vehicle is set at 30 km/h, the average delivery cost is 50 CNY/km, the vehicle dispatch cost is 200 CNY/vehicle, and the service time at customer points is 2 minutes. The earliest time the user can tolerate is 10 minutes at the left end of the time window, and the latest is 15 minutes at the right end of the time window. The penalty coefficient for customer satisfaction is 5000. The maximum load capacity of the vehicle is 100 kg.

This paper selects the traffic congestion data of Beijing's road network during the working days in February 2021 from the Baidu map traffic congestion real-time monitoring platform. Based on the simulation data of the speed change in the time-dependent road network in this design, the length of the time period is set to 10 minutes in the peak period and 30 minutes in the off-peak period. At the same time, for the convenience of the experiment, it is assumed that the road types in the road network are roughly the same, and the speed change laws are the same. Based on the above assumptions, the speed change data under the time-dependent road network described in this paper are shown in Table 2.

All experiments in this section are written based on the software Python 3.9 and run on a macOS system with a CPU configuration of 2.6 GHz six-core Intel Core i7. The parameters required for the experiment are set as follows: the population size is 50, the maximum number of iterations is 400, the crossover probability is 0.85, the mutation probability is 0.05, and the parameter *G*_max_ in adaptive variable neighborhood search is 20.

### 4.1. Algorithm Performance Verification

**Figure 6:**
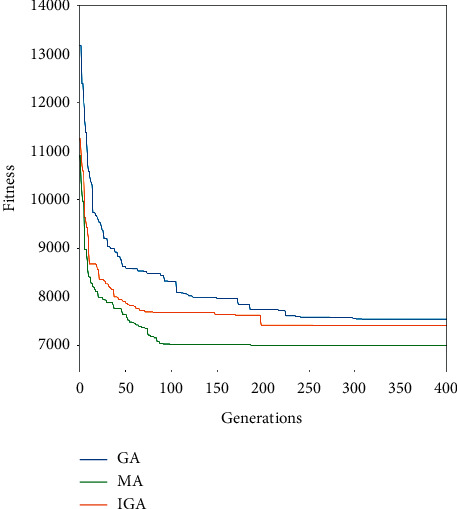
Comparison of algorithm iteration process.

The algorithm comparison part chooses to compare the memetic algorithm with the greedy initialization genetic algorithm and the basic genetic algorithm. The basic genetic algorithm uses a random method to initialize the population, and the greedy genetic algorithm uses the greedy strategy to generate the initial population. The number of populations in the three algorithms, crossover rate, and other parameters are consistent with the memetic algorithm. The comparison diagram of the iterative process of the three algorithms is shown in Figure 6.

Figure 6 shows that since both the IGA and MA use the greedy algorithm to generate the initial solution, the convergence curves of these two algorithm pairs show that their initial population is significantly better than that of the GA that generates the initial solution in a random way. In the iterative process of the memetic algorithm, the in-depth exploration of the variable neighborhood strategy makes the algorithm rapidly decline and converge to a better level at the beginning of the iteration, and it is significantly better than the other two algorithms in terms of convergence speed and solution effect. The validity of the memetic algorithm was verified.

### 4.2. Difference Analysis of Congestion Period

The departure times are set at 18:00 and 12:00, respectively, corresponding to the evening peak hours and the peak hours, and each experiment runs 30 times. The optimal results are shown in Table 3.

**Figure 7:**
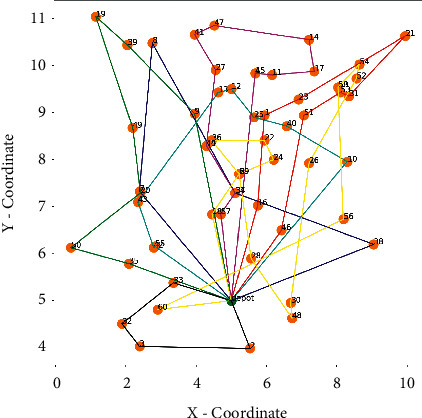
Schematic diagram of the route when the departure time is 18.

**Figure 8:**
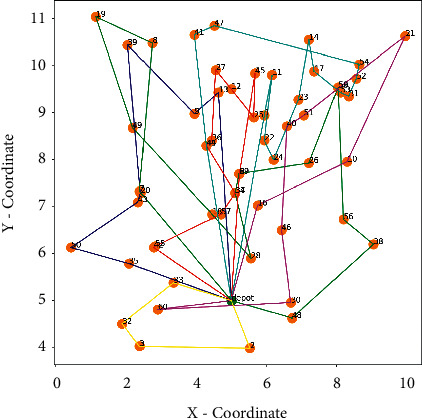
Schematic diagram of the route when the departure time is 12.

The distribution scheme and route diagram corresponding to the results are shown in Figure 7 and Figure 8, and the optimal results are shown in Table 4.

It can be seen from Table 3 that the delivery route departure during the evening peak period costs 563.88 CNY more than that during the normal peak period, the total driving distance during the off-peak period is 5.5 km less than that in the evening peak period, and the customer satisfaction penalty cost in the off-peak period is 288.88 CNY less than that in the evening peak period. It can be seen that driving at a low speed during peak hours will increase the total driving distance of the vehicle and at the same time will bring greater penalty costs, resulting in a decrease in the user's time satisfaction. Therefore, when carrying out route planning, the influence of different congestion periods on vehicles should be taken into account in the route planning scheme. Methods such as adding vehicles during peak hours or doing promotions during off-peak hours should be adopted to meet customers' time window needs, improve customer satisfaction, and divert orders from peak hours to off-peak hours for delivery.

### 4.3. Sensitivity Analysis

**Figure 9:**
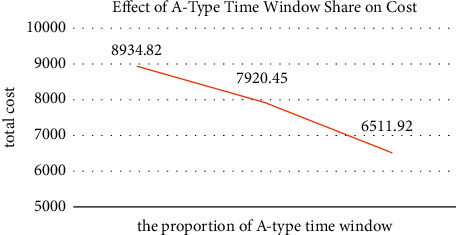
Changes in total delivery cost under different proportions of A-type time window.

The customer's time window is related to the satisfaction factor, which in turn has an impact on the total cost. To analyze the influence of the proportions of the two time windows on the distribution cost, the time window of the original example was modified. In the original calculation example, the two types of time windows of A and B account for 50% each. If the left end of the time window of all nodes in the calculation example is changed to 0, then the proportion of type A customers is 100%. If the left end of the time window of all nodes in the calculation example is changed to the right end of the time window minus 10 minutes, then the proportion of type A customers is 0%. The impact of the three proportions on cost is shown in Figure 9.

The result analysis of the three proportions of time window shows that due to the loose characteristics of the A-type time window, in the case with the larger A-type time window proportion, the total cost of distribution is smaller, and the penalty cost decreases more obviously. As shown in Figure 9, when the A-type time window accounts for 0%, the total cost is larger than that in the case of 50%. When the A-type time window accounts for 100%, the total cost decreases significantly, and at this time, the average customer satisfaction in the results of multiple experiments can reach 100%. Therefore, when providing delivery services to different types of customers, enterprises should pay more attention to the time window requirements of designated customers and can reasonably plan paths by assigning greater weight to such orders.

**Figure 10:**
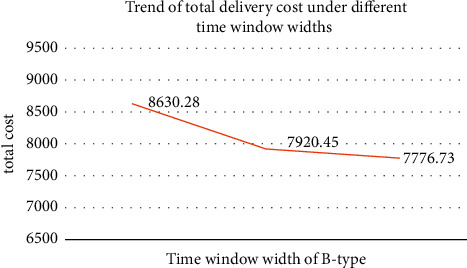
Changes in total delivery cost under different time window widths of B type.

In the calculation example, the width of the B-type time window is 20 minutes. To analyze the influence of the width of the time window on the cost, a wider and narrower time window than the original calculation example are designed as an experimental calculation example. In the case of narrow time window, both ends of the time window in the original case are compressed by 5 minutes, and in the case of wide time window, both ends of the time window in the original case are expanded by 5 minutes.  Figure 10 shows the trend of cost changes under the three time window widths.

As can be seen from Figure 10, when the time window is shortened, in order to meet the strict delivery time expectations of customers, the total cost increases significantly. And the impact of narrowing the time window on the cost is more obvious. In addition, widened time window will reduce the cost to a certain extent, but when the length of the time window reaches a certain level, the increase in the time window has less impact on the reduction of cost, and it is difficult to bring about a big change in the result. Therefore, enterprises can obtain higher customer satisfaction by increasing the time window width that can be specified for type B orders. However, it should be noted that when the time window width reaches a certain level, the total cost will be reduced due to the consideration of driving cost and vehicle dispatch cost. Hence, setting a reasonable window width should take into account both cost and customer satisfaction.

## 5. Conclusion

This paper considers the phenomenon that instant delivery is affected by external traffic conditions, and proposes a route planning model with different customer time window types under the time-dependent road network. In addition, the memetic algorithm based on genetic algorithm and variable neighborhood strategy is designed to solve the model, and the rationality and effectiveness of the model and algorithm are verified by experiments. At the same time, this paper constructs the corresponding satisfaction function according to the different customers' demand for arrival time, which is more in line with the actual consumption scene of instant delivery. The routing schedule of instant delivery distribution on time-dependent network takes the regular traffic congestion as the direct influence factor of vehicle travel time, which provides a method for instant delivery distribution to consider the external environment for route planning. In addition, there are phenomena such as excess orders during peak hours and capacity runs during the epidemic period in the instant delivery industry, which in turn cause an imbalance in capacity matching and affect delivery efficiency. Therefore, considering multisite joint distribution in the region or introducing unmanned equipment for multistage distribution is worthy of further study.

## Figures and Tables

**Table 1:** Relevant data of the example.

Pickup nodes	Pickup node coordinates	Delivery nodes	Delivery node coordinates	Demand (kg)	*e*_*i*_	*l*_*i*_
0	[5.00, 5.00]	0	[5.00, 5.00]	-	0	90
1	[5.94, 8.94]	31	[8.34, 9.35]	23	0	10
2	[5.53, 3.99]	32	[1.89, 4.51]	14	0	10
3	[2.38, 4.03]	33	[3.35, 5.38]	16	0	10
4	[5.68, 9.84]	34	[5.11, 7.29]	32	0	10
5	[3.95, 8.98]	35	[2.08, 5.79]	10	0	10
6	[5.23, 7.70]	36	[4.44, 8.41]	20	0	20
7	[2.38, 7.33]	37	[5.11, 7.29]	33	0	20
8	[2.75, 10.48]	38	[9.05, 6.20]	10	0	20
9	[3.95, 8.98]	39	[2.04, 10.44]	30	0	20
10	[8.30, 7.96]	40	[6.58, 8.71]	28	0	20
11	[6.16, 9.80]	41	[3.95, 10.66]	40	0	30
12	[5.00, 9.50]	42	[2.79, 6.13]	40	0	30
13	[4.63, 9.43]	43	[2.34, 7.10]	20	0	30
14	[7.21, 10.55]	44	[4.29, 8.30]	22	0	30
15	[5.68, 9.84]	45	[4.3, 8.29]	20	0	30
16	[5.75, 7.03]	46	[6.43, 6.50]	20	10	30
17	[7.36, 9.88]	47	[4.51, 10.85]	10	10	30
18	[4.45, 6.84]	48	[6.73, 4.63]	21	10	30
19	[1.14, 11.04]	49	[2.19, 8.68]	20	10	30
20	[2.41, 7.29]	50	[0.43, 6.13]	43	10	30
21	[9.95, 10.63]	51	[7.06, 8.94]	15	20	40
22	[5.94, 8.41]	52	[8.56, 9.73]	40	20	40
23	[6.91, 9.28]	53	[8.11, 9.43]	10	20	40
24	[6.20, 8.00]	54	[8.64, 10.03]	10	20	40
25	[5.64, 8.90]	55	[2.83, 6.16]	26	20	40
26	[7.21, 7.93]	56	[8.19, 6.73]	10	30	50
27	[4.55, 9.91]	57	[4.70, 6.84]	25	30	50
28	[5.56, 5.90]	58	[8.04, 9.54]	20	30	50
29	[5.22, 7.70]	59	[8.07, 9.54]	41	30	50
30	[6.69, 4.96]	60	[2.90, 4.81]	20	30	50

**Table 2:** Vehicle speed in each period of service period.

Time period	Speed (km/h)	Time period	Speed (km/h)
12:00–12:30	26.57	17:40–17:50	17.57
12:30–13:00	26.75	17:50–18:00	16.67
13:00–13:30	26.28	18:00–18:10	16.21
13:30–14:00	25.4	18:10–18:20	16.02
14:00–14:30	24.9	18:20–18:30	16.03
14:30–15:00	24.8	18:30–18:40	16.38
15:00–15:30	24.78	18:40–18:50	17.03
15:30–16:00	24.87	18:50–19:00	17.88
16:00–16:30	24.79	19:00–19:30	19.87
16:30–17:00	23.77	19:30–20:00	23.09
17:00–17:10	22.36	20:00–20:30	25.01
17:10–17:20	20.93	20:30–21:00	25.71
17:20–17:30	19.55	21:00–21:30	25.99
17:30–17:40	18.56	21:30–22:00	26.28

**Table 3:** Total cost and cost composition at different departure times.

Departure time	Total cost (CNY)	Number of vehicles (vehicles)	Total driving distance (km)	Distance cost (CNY)	Penalty cost (CNY)
18:00	7920.45	6	117.50	5875	845.45
12:00	7356.57	6	110.00	5600	556.57

**Table 4:** Delivery plans at different departure times.

Departure time	Delivery plan
18:00	0-17-14-11-47-41-8-19-49-44-26-38-56-0
	0-5-27-12-13-7-20-43-42-50-35-57-37-0
	0-4-15-25-9-39-45-34-30-60-55-0
	0-16-6-29-36-18-28-48-46-24-58-54-59-0
	0-1-22-31-10-40-23-21-52-53-51-0
	0-2-3-32-33-0

12:00	0-25-5–27-12-13-20-43-50-35-42-55-57-0
	0-10-24-22-40-23-54-21-52-53-51-0
	0-1-31-17-14-11-15-47-41-44-45-0
	0-9-8-39-19-49-26-38-56-0
	0-4-6-34-36-18-28-48-30-60-29-59-58-0
	0-2-3-32-33-7-37-16-46-0

## Data Availability

The data used to support the findings of this study are available from the corresponding author upon request.
